# Supplementary material for: The glucuronyltransferase B4GAT1 is required for initiation of LARGE-mediated α-dystroglycan functional glycosylation
Source: eLife. 2014 Oct 3;3:e03941. doi: 10.7554/eLife.03941 (PMC4227050; doi:10.7554/eLife.03941)
Supplement: Figure 3—source data 1. — Chemical shifts (ppm) of the signals in the 1H and 13C NMR spectra of the enzymatic reaction product of GlcA-β1,4-Xyl-β-MU of the glycosyltransferase B4GAT1. DOI: http://dx.doi.org/10.7554/eLife.03941.008 [file elife03941s001.docx]

**Figure 3 - source data 1**  Chemical shifts (ppm) of the signals in the ^1^H and ^13^C NMR spectra of the enzymatic reaction product of GlcA-β1,4-Xyl-β-MU of the glycosyltransferase B4GAT1.

^1^H/^13^C (ppm)^a^ _________________________________________________________________________

Products Sugar Aromatic Ring^b^

_________________________________________ ______________________________

1 2 3 4 5 6 3 4-CH_3_ 6 7 9

→4)-β-D-Xyl-MU 5.21 3.64 3.72 3.93 4.18, 3.61 6.28 2.45 7.76 7.13 7.12

**A** 102.7 75.2 76.3 78.9 65.8 114.0 20.6 129.4 116.6 106.3

β-D-GlcA-(1→ 4.55 3.32 3.50 3.51 3.73

**B** 103.6 75.4 78.1 74.4 78.3 178.5^b^

^a^ Chemical shifts at 25°C in 10 mM sodium phosphate, pH 6.5.

^b^ Assigned based on the overlay of HMQC and HMBC spectra.
