# Supplementary material for: The glucuronyltransferase B4GAT1 is required for initiation of LARGE-mediated α-dystroglycan functional glycosylation
Source: eLife. 2014 Oct 3;3:e03941. doi: 10.7554/eLife.03941 (PMC4227050; doi:10.7554/eLife.03941)
Supplement: Figure 7—source data 1. — Chemical shifts (ppm) of the signals in the 1H and 13C NMR spectra of the tetrasaccharide of GlcA-β1,3-Xyl-α1,3-GlcA-β1,4-Xyl-β-MU produced by B4GAT1 and LARGE. DOI: http://dx.doi.org/10.7554/eLife.03941.018 [file elife03941s002.docx]

**Figure 7 - source data 1.** Chemical shifts (ppm) of the signals in the ^1^H and ^13^C NMR spectra of the tetrasaccharide of GlcA-β1,3-Xyl-α1,3-GlcA-β1,4-Xyl-β-MU produced by B4GAT1 and LARGE.

^1^H/^13^C (ppm)^a^ _________________________________________________________________________

Products Sugar Aromatic Ring^b^

_________________________________________ ______________________________

1 2 3 4 5 6 3 4-CH_3_ 6 7 9

→4)-β-D-Xyl-MU 5.22 3.64 3.73 3.93 4.18, 3.60 6.30 2.47 7.78 7.14 7.15

**A** 102.7 75.2 76.2 79.1 65.8 114.0 20.6 129.4 116.5 106.4

→3)-β-D-GlcA-(1→ 4.56 3.42 3.63 3.74 3.73

**B** 103.9 74.0 83.4 75.0 78.3 178.5^b^

→3)-α-D-Xyl-(1→ 5.34 3.71 3.85 3.68 3.68, 3.92

**C** 101.3 73.7 84.8 70.8 63.8

β-D-GlcA-(1→ 4.69 3.37 3.53 3.51 3.74

**D** 105.2 76.0 78.1 74.4 78.3 178.5^b^

^a^ Chemical shifts at 25°C in 10 mM sodium phosphate, pH 6.5.

^b^ Assigned based on the overlay of HMQC and HMBC spectra.
